# Herkogamy and Its Effects on Mating Patterns in *Arabidopsis thaliana*

**DOI:** 10.1371/journal.pone.0057902

**Published:** 2013-02-26

**Authors:** Yonghai Luo, Alex Widmer

**Affiliations:** ETH Zurich, Plant Ecological Genetics, Institute of Integrative Biology (IBZ), Zurich, Switzerland; Universidad Miguel Hernández de Elche, Spain

## Abstract

The evolution of mating systems, which exhibit an extraordinary diversity in flowering plants, is of central interest in plant biology. Herkogamy, the spatial separation of sexual organs within flowers, is a widespread floral mechanism that is thought to be an adaptive trait reducing self-pollination in hermaphroditic plants. In contrast with previous studies of herkogamy that focused on plants with relatively large floral displays, we here characterized herkogamy in *Arabidopsis thaliana*, a model plant with a strong selfing syndrome. Developmental features, reproductive consequences, and genetic architecture of herkogamy were exploited using naturally variable *A. thaliana* accessions, under both greenhouse and natural conditions. Our results demonstrate that the degree of herkogamy can strongly influence the mating patterns of *A. thaliana*: approach herkogamy can effectively promote outcrossing, no herkogamy is also capable of enhancing the opportunity for outcrossing, and reverse herkogamy facilitates efficient self-pollination. In addition, we found that the expression of herkogamy in *A. thaliana* was environment-dependent and regulated by multiple quantitative trait loci. This study reveals how minor modifications in floral morphology may cause dramatic changes in plant mating patterns, provides new insights into the function of herkogamy, and suggests the way for dissecting the genetic basis of this important character in a model plant.

## Introduction

Plant mating systems are highly diverse and have profound effects on reproductive success under variable ecological conditions. Self-fertilization (selfing) may be adaptive when mates or pollinators are limited, as originally proposed by Darwin, because it confers reproductive assurance [1]. This advantage may be offset by inbreeding depression, the reduced fitness of offspring in comparison to outcrossed progeny [1]–[3]. There are several obvious advantages of outcrossing, including avoidance of inbreeding depression by reducing the expression of deleterious recessive mutations, increased potential for rapid adaptation to changing ecological conditions, and maintenance of long-term adaptive capacity during evolution [1]–[5]. As a consequence, most plants have evolved a wide variety of physiological or morphological mechanisms to ensure or facilitate outcrossing [1], [2], [6].

Herkogamy, the spatial separation of male and female reproductive organs within flowers, is almost ubiquitous in animal-pollinated hermaphroditic plants. In self-incompatible species, herkogamy may function primarily to reduce sexual interference, whereas in self-compatible plants, it is usually considered an adaptive character that decreases the likelihood of self-pollination and increases the opportunity for outcrossing [6], [7]. Indeed, a number of studies have reported evidence for a monotonically increasing relationship between the degree of herkogamy and outcrossing rate across diverse plant species, including *Nicotiana* [8], *Clarkia* [9], *Turnera* [10], *Mimulus* [11], *Aquilegia* [12], and *Datura* [13], although there are exceptions (e.g., *Narcissus*, [14], [15]). Notably, these self-compatible plant species are commonly with relatively large floral displays and, in these cases, the role of herkogamy in promoting outcrossing may be complex. On the one hand, large floral displays can increase outcrossing rates, probably through enhanced pollinator attraction [16]–[18]. On the other hand, large floral displays could promote geitonogamy, self-pollination as a consequence of pollen dispersal among flowers on the same plant [12], [19], [20]. To clarify the role of herkogamy in promoting outcrossing, ideally it requires a highly selfing species with phenotypic variation in herkogamy and with a small floral display.

Herkogamy demonstrates moderate to high heritability across different plant species and may be able to respond to selection rapidly [13], [21]–[23]. The molecular basis of herkogamy, however, remains largely unexplored across diverse species (but see [24]). This may be primarily attributable to the limited genomic resources that are available for those plant species, which are generally non-model organisms. Hence, studying ecologically important and complex characters (e.g., herkogamy) in model organisms such as *Arabidopsis thaliana* may be particularly beneficial.

**Figure 1:**
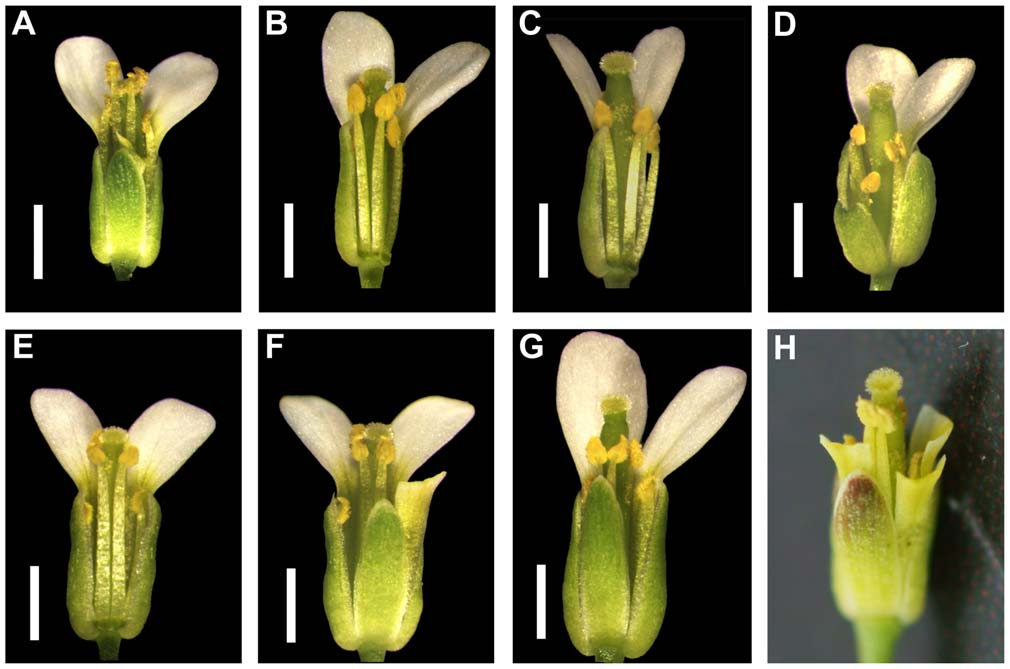
Phenotypic variation in herkogamy in different *A. thaliana* genotypes. Freshly opened flowers of Col-0 (**A**), weak phenotype of BRA (**B**), strong phenotype of BRA (**C**), SIM (**D**), F_1_ hybrid of BRA and Col-0 (**E**), F_1_ hybrid of SIM and Col-0 (**F**), and F_1_ hybrid of SIM and BRA (**G**) under greenhouse conditions. Scale bar  = 1 mm. (**H**) A flower of SIM showing weak approach herkogamy under natural conditions at high altitude. Parts of petals and sepals were removed to improve visibility in some flowers.

*A. thaliana* is a model plant used for a wealth of studies, ranging from molecular genetics and genomics to evolution and ecology [25], [26]. It is a predominantly selfing species with a strong selfing syndrome – a characteristic set of floral morphology and function (e.g., small, unscented, and inconspicuous flowers without effective separation of sexual organs) that facilitates self-pollination [1], [27], [28]. Typically estimated outcrossing rates ranged from 0.3% to 2.5% [29]–[34], but could exceed 10% in some populations [33], [34]. The size of a mature *A. thaliana* flower is as small as 2–3 mm and anthers are typically positioned closely above the stigma (Figure 1A). This arrangement is known as reverse herkogamy [6]. Approach herkogamy, in which the stigma is positioned above the anthers, has not been reported to date in natural *A. thaliana* accessions.

In this study we demontrate the existence of approach herkogamy in natural *A. thaliana* accessions collected from alpine regions in Europe and Africa and elucidate the function of herkogamy. Specifically, we address the following scientific questions: (1) What is the phenotypic variation and plasticity in herkogamy in *A. thaliana*? (2) What is the reproductive consequence of the degree of herkogamy in this highly selfing plant species under different environmental conditions? (3) What is the genetic architecture underlying this complex trait?

## Results

### Characterization of approach herkogamy under greenhouse conditions

**Figure 2:**
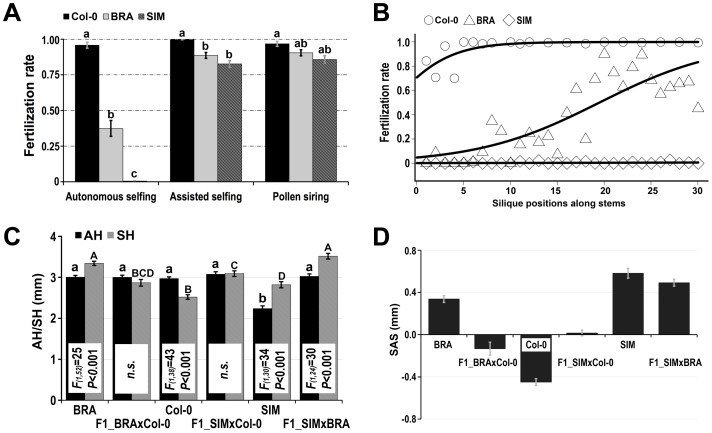
Characterization of approach herkogamy in the greenhouse. (**A**) Means with standard errors (SEs) of fertilization rates in Col-0, BRA, and SIM – upon autonomous selfing, assisted selfing, and pollination of a male-sterile line (pollen siring). Letters above bars indicate significant differences (Tukey's post-hoc significance tests, α = 0.05) within each fertilizing group. (**B**) Self-fertilization rates in successive siliques along the main stems of Col-0, BRA, and SIM. The regression function is: y  =  exp(a+b*x)/(1+exp(a+b*x)). Coefficients (a/b) of non-linear models are 0.89/0.33, −2.98/0.15 and −5.97/0.035 for Col-0, BRA and SIM, respectively. (**C**) Measurements of anther height (AH) and stigma height (SH) in flowers of Col-0 (flowers N = 20), BRA (N = 27), and SIM (N = 16) and their hybrids (F1_BRAxCol-0, N = 16; F1_SIMxCol-0, N = 20; F1_SIMxBRA, N = 13). Significant differences (one-way ANOVA) between AH and SH within each genotype are indicated in the white boxes. Letters above bars indicate statistically significant differences (Tukey's post-hoc significance tests, α = 0.05) in AH or SH across all examined accessions. (**D**) Calculated SAS (SAS  =  SH – AH; means± SEs) of Col-0, BRA, SIM and their hybrids.

In an analysis of natural *A. thaliana* accessions collected from alpine areas in Europe and Africa (Table S1), we observed substantial variation in self-fertilization rates (ranging from ∼0 to ∼100%) under pollinator-free greenhouse conditions. In particular, two of the investigated accessions, called BRA and SIM, exhibited strongly reduced seed set in comparison to the lab standard line Columbia (Col-0; Figure 2A). BRA showed a gradient of increasing spontaneous self-fertilization throughout the inflorescence and SIM largely failed to set seeds autonomously, while Col-0 displayed incomplete self-fertilization only in the first flowers along the main stem (Figure 2B). Analyses of floral morphology revealed that, in contrast to Col-0, BRA and SIM displayed approach herkogamy in the greenhouse (Figures 1A–D). Moreover, we observed phenotypic variation in the degree of approach herkogamy in BRA individuals (Figures 1B & 1C), while the phenotype was highly stable in SIM (Figure 1D).

To test whether reduced spontaneous self-fertilization is due to deficiencies in sexual functions such as male or female sterility, we manually pollinated flowers with their self pollen (assisted selfing) and used the pollen to pollinate flowers of a male-sterile line (pollen siring). The resulting fertilization rates were high (>80%) for all examined accessions (Figure 2A), indicating that neither male nor female sterility can account for the strongly reduced spontaneous self-fertilization. Moreover, the success of assisted selfing excluded the possibility of dichogamy, the temporal separation of the sexes. These results suggest that the observed approach herkogamy is likely the major factor underlying the strongly reduced spontaneous self-fertilization rates in BRA and SIM under greenhouse conditions.

We measured anther height and stigma height in Col-0, BRA, and SIM in the greenhouse (Figure S1A). In BRA, the anther height did not differ from Col-0 (*P* = 0.996), whereas the stigma height was significantly increased in comparison to Col-0 (*P*<0.001; Figure 2C). In contrast, SIM had significantly reduced anther height (*P*<0.001) but increased stigma height than Col-0 (*P*<0.05; Figure 2C). We then quantified the degree of separation between anthers and stigmas (SAS) as the difference between stigma height and anther height (i.e., SAS  =  stigma height – anther height), and found that both BRA and SIM had positive SAS values (i.e., approach herkogamy), whereas Col-0 had a negative SAS value (i.e., reverse herkogamy; Figure 2D). Hybrids between Col-0 and either BRA or SIM exhibited similar anther or stigma height, and thus had SAS values close to zero (i.e., no herkogamy; Figures 1E, 1F, 2C & 2D). Hybrids between BRA and SIM resembled the strong phenotype of approach herkogamy in BRA (Figures 1G, 2C & 2D), suggesting that BRA alleles are dominant. Developmental differences in floral organs between BRA, SIM, and hybrids suggest that different genetic networks may control approach herkogamy in these two accessions.

### Herkogamy and its effects on mating patterns under natural conditions

**Figure 3:**
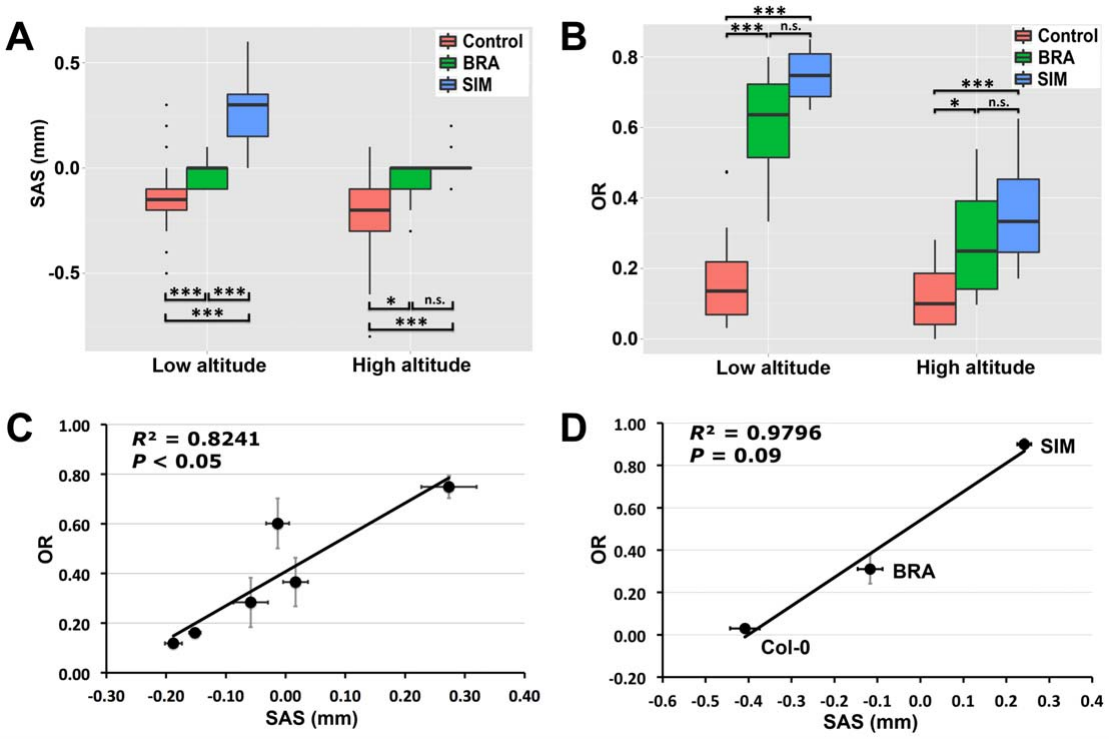
Herkogamy and its reproductive consequence under natural conditions. Boxplots of SAS (the proxy of herkogamy, **A**) and outcrossing rates (OR, **B**) in BRA, SIM, and the control accessions growing in the field experiments (high and low altitudes). Significant differences are indicated. ***, *P*<0.001; *, *P*<0.05; n.s., *P*>0.05. Scatterplots and Pearson's correlation between SAS and OR in the field experiments (**C**) and the common-garden experiment (**D**). Means with standard errors are shown.

To assess the effects of approach herkogamy on mating patterns in BRA and SIM under natural conditions, we conducted field experiments in which we grew BRA and SIM together with 13 control accessions that exhibit reverse herkogamy (Table S1). Because both BRA and SIM were originally collected at high altitudes, we considered whether the expression of approach herkogamy in these two accessions was related to climatic conditions at different altitudes, and therefore grew one experimental population at high altitude and one at low altitude. Since each of the 13 control accessions displayed reverse herkagomy and was different from both BRA and SIM, we grouped them as control for comparison. Overall, substantial variation in SAS (a proxy for herkogamy) and outcrossing rates were observed within and among accessions as well as field sites (Figures 3A & 3B). Firstly, the outcrossing rates detected in our experimental populations were high and variable. We randomly chose 3–4 test plants from each accession out of each field site for estimating their outcrossing rates. In total, we identified 717 outcrossed offspring from the 3502 progeny that were genotyped using molecular markers. The average outcrossing rates for the populations were 22.39% (low altitude) and 14.57% (high altitude), and the accession means ranged from 4.17% to 74.87% (low altitude) and from 2.81% to 36.81% (high altitude). Secondly, at low altitude, SIM exhibited approach herkogamy (i.e., positive SAS) and had the highest outcrossing rate. At high altitude, however, the degree of approach herkogamy in SIM was highly reduced and most flowers expressed no herkogamy (i.e., SAS values close to zero; but see Figure 1H). Accession BRA, in contrast, did not express approach herkogamy at either high or low altitude, but instead had no herkogamy. Nevertheless, both BRA and SIM had higher outcrossing rates than the control accessions. This indicates that an absence of reverse herkogamy may reduce the efficiency of self-pollination and thus enhances the opportunity for cross-pollination in nature. Thirdly, at both high and low altitude, the control accessions stably expressed reverse herkogamy (i.e., negative SAS) and had significantly lower outcrossing rates in comparison to BRA and SIM. This suggests that efficient self-pollination may be a benefit of reverse herkogamy in this small plant with a pronounced selfing syndrome. Importantly, the degree of herkogamy expressed under field conditions was highly correlated with outcrossing rates (*R* ^2^ = 0.82; Figure 3C), suggesting a causal relationship.

A further common-garden experiment that was carried out in a rural field site with only three accessions (i.e., Col-0, BRA, and SIM) consistently revealed a positive and linear relationship between the degree of herkogamy and outcrossing rates (Figure 3D). These results together indicate that the degree of herkogamy has a strong effect on mating patterns in *A. thaliana*: approach herkogamy can effectively promote outcrossing, no herkogamy may facilitate outcrossing, and reverse herkogamy likely ensures efficient autonomous self-pollination.

### Expression of herkogamy is temperature sensitive

**Figure 4:**
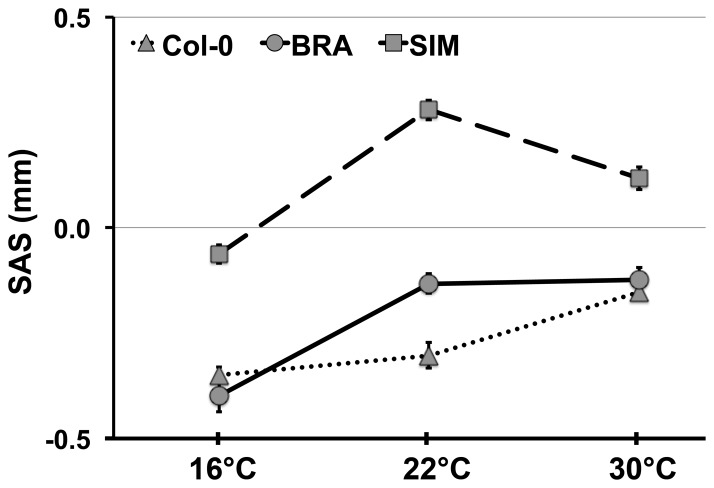
The influence of ambient temperature on the expression of herkogamy. Accession means of SAS (the proxy of herkogamy) in Col-0, BRA, and SIM plants grown under different ambient temperatures and identical light conditions are shown.

We observed both phenotypic variation and plasticity in herkogamy in our field experiments (Figure 3A). Because temperature changes substantially with increasing altitude, we experimentally tested the influence of ambient temperature on the expression of herokogamy. For this purpose, we grew Col-0, BRA, and SIM plants in climate chambers that differ only in ambient temperature and estimated SAS. Highly significant genotype by temperature interactions were found (two-way ANOVA, *F_4,71_*  = 12, *P*<0.001). At 22°C or 30°C, SIM displayed approach herkogamy (i.e., positive SAS) and set few seeds autonomously, whereas BRA had negative SAS and set seeds, similar to Col-0. At 16°C, accessions SIM, BRA, and Col-0 displayed reverse herkogamy and produced full seed set through autonomous self-pollination (Figure 4). These findings indicate that the expression of herkogamy in BRA and SIM is highly temperature dependent, and the fact that no approach herkogamy in BRA was found in this experiment indicates the influence of other environmental factors in this accession.

### Genetic architecture underlying approach herkogamy

**Figure 5:**
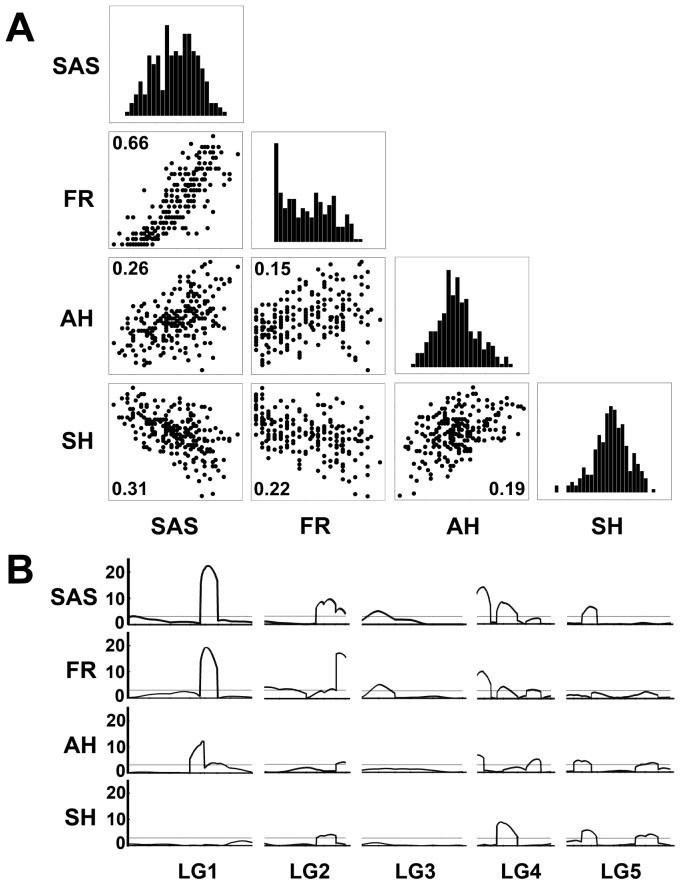
Quantitative trait locus (QTL) analysis of approach herkogamy. (**A**) Distributions and correlations of untransformed phenotypic data of SAS (the proxy of herkogamy), self-fertilization rate (FR), anther height (AH), and stigma height (SH) in an F2 population derived from a cross between SIM and Col-0. The R^2^ values based on Pearson's correlation are shown in the plots (all *P*<0.001). (**B**) Alignment of logarithm of odds (LOD) maps for SAS, FR, AH, and SH. The dashed lines indicate the genome-wide significance threshold at LOD 3.0. LG, linkage group.

To investigate the genetic architecture underlying approach herkogamy we performed segregation analysis and QTL mapping in an F_2_ population derived from a cross between SIM and Col-0 in the greenhouse. We analyzed four traits – anther height, stigma height, self-fertilization rate, and SAS – in 227 F_2_ individuals and found that self-fertilization rates were positively correlated with SAS (*R* ^2^ = 0.66; Figure 5A). In total, we identified 9 QTLs underlying SAS that were distributed across all 5 chromosomes (Figure 5B). Each QTL explained between 2% and 18% of the variance, for a total of 55% of the variance in SAS. The QTLs controlling self-fertilization rate were largely the same as those underlying SAS, suggesting a common genetic basis and thus a causal relationship (Figure 5B). QTLs affecting anther height and stigma height represented a subset of those underlying SAS, in an additive pattern, indicating that anther height and stigma height are independently controlled and collectively confer approach herkogamy in SIM (Figure 5B).

## Discussion

### Natural genetic variation in outcrossing in *A. thaliana*

Given that *A. thaliana* is generally considered to be a highly selfing plant, the outcrossing rates detected in our experimental populations were surprisingly high and variable. As we cannot recognize outcrossing events when the pollen donor has the same genotypes as the pollen recipient at the examined loci, the detected outcrossing rates may be conservative. In nature, many factors can influence outcrossing rates in a population, such as population density, pollinator availability, and floral morphology. It remains unknown whether some *A. thaliana* populations exhibit such high outcrossing rates in nature as observed in our experimental populations. Therefore, it would be interesting to examine the outcrossing rates of these *A. thaliana* populations (i.e., BRA and SIM) in their native habitats, ideally over several continuous growth seasons. However, outcrossing events might go undetected if one examines only a limited number of genetic loci, because of insufficient genetic variation within natural populations of *A. thaliana*; a colonizing species usually characterized by small local population sizes and strong population structure [33], [35]. Accordingly, in this study we designed experimental arrays consisting of multiple *A. thaliana* genotypes in random arrangements that facilitated the detection of outcrossed progeny through marker-assisted analyses.

Nevertheless, the remarkable variation in outcrossing rates among accessions within each experimental population cannot simply be attributed to external factors, and thus indicates that natural genetic variation among the studied accessions has a major effect on outcrossing. Even in the high-altitude area (alpine climatic conditions), the outcrossing rates in two alpine accessions (BRA and SIM) were high (Figure 3B). Therefore, it may be possible that substantial outcrossing events occur in their native populations. The existence of “outcrossing hotspots” in *A. thaliana* observed in a previous study reinforces our speculation [34]. Hence, we consider that mixed mating in natural *A. thaliana* populations may be more common than has been acknowledged to date.

### Herkogamy and its reproductive consequences

**Figure 6:**
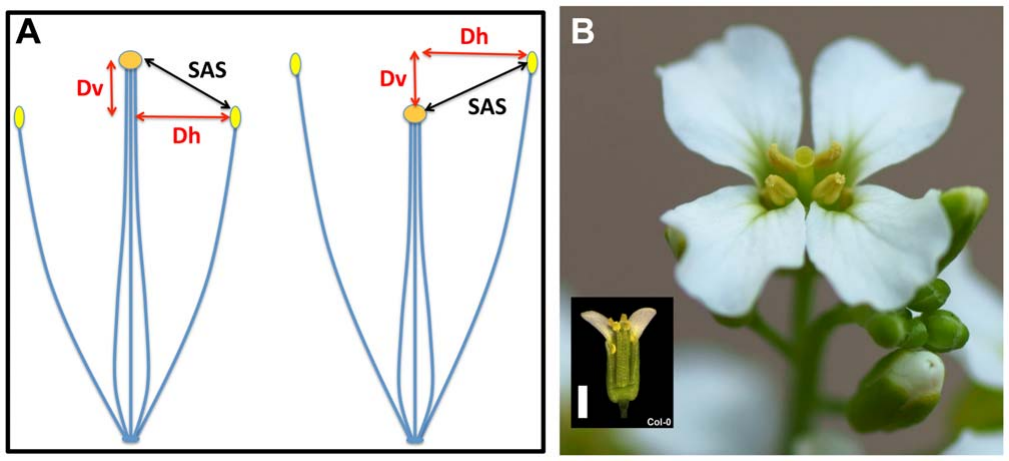
Components of herkogamy. (**A**) Diagrams for approach herkogamy (left) and reverse herkogamy (right). Theoretically, SAS (the proxy of herkogamy) is determined by two main components, the vertical (Dv) and horizontal separation (Dh) between stigma and anther. (**B**) A representative flower of *A. lyrata* showing considerable separation between stigma and anther in both vertical and horizontal directions. The inset shows an *A. thaliana* Col-0 flower for comparison (parts of petals and sepals were removed to improve visibility). Scale bar  = 1 mm.

The main components of herkogamy in most flowering plants can be divided into vertical and horizontal separation of stigmas and anthers within flowers (Figure 6A). Two types of herkogamy that focus on the vertical separation, approach and reverse herkogamy, as well as their reproductive consequence have been well investigated in diverse plant species (see the review in [6]). The functional relevance of the horizontal separation, in contrast, has received less attention. In *A. thaliana*, horizontal stigma-anther separation has been lost, most likely during the evolution of the selfing syndrome, in contrast to its outcrossing relatives (e.g., *A. lyrata*; Figure 6B). As a consequence, ripe pollen in a normal *A. thaliana* flower can be efficiently deposited on the receptive stigma by gravity for self-pollination. This could be quite different to those self-compatible plants with relatively large floral displays, in which there is horizontal separation to some degree, and thus is an important point when considering the function of herkogamy.

In this study, we included a relatively large number of control accessions with different origins representing 10 natural accessions collected in the same areas as BRA, together with another high altitude accession (Sha), the lab standard line (Col-0), and another widely used accession (Bay-0). We found that all control accessions displayed reverse herkogamy and had relatively low outcrossing rates in our experiments, compared to the high outcrossing rates in accessions BRA and SIM (Figures 3A & 3B). These results indicate that reverse herkogamy facilitates efficient self-pollination in the absence of horizontal stigma-anther separation, whereas approach herkogamy (or no herkogamy) can reduce the efficiency of self-pollination and thus promote outcrossing in *A. thaliana*. Importantly, the observed change in the degree of herkogamy in *A. thaliana* was minute in the investigated accessions and SAS values typically ranged from −0.5 mm to 0.5 mm (Figures 2D & 3A). Nevertheless, these subtle floral modifications caused a dramatic change in the mating patterns of these *A. thaliana* accessions.

### Possible significance of environment-dependent herkogamy

The expression of approach herkogamy in accessions BRA and SIM was highly environment-dependent. What is the ecological significance of such a trait? We hypothesize that the environment-dependency of approach herkogamy in *A. thaliana* may be adaptive and thus maintained by natural selection if approach herkogamy is expressed under suitable ecological conditions and confers the advantages of outcrossing, while it is repressed under less suitable conditions, such as during cold and rainy weather periods, when pollinators are scarce. A previous study on *Collinsia verna* showed that populations had a dynamic mating system, adapting to different pollination environments, which resulted in mixed mating and reproductive assurance [36]. Together, these findings suggest that environment-dependent flexible mating systems may be advantageous.

### Molecular basis of approach herkogamy

Approach herkogamy has previously been reported in laboratory mutants of *A. thaliana* such as *opr3*, *hth* and *rdr6*, where it is usually associated with reduced fertility [37]–[39]. The natural examples of approach herkogamy analyzed in the present study are different from these reported single-gene mutants. Our natural accessions displaying environment-dependent approach herkogamy have normal fecundity and approach herkogamy in SIM is controlled by multiple QTLs, similar to the situation reported in other species [24], [40], [41]. Moreover, BRA and SIM were collected from disjunct geographical regions in the Swiss and Ethiopian mountains and approach herkogamy is achieved differently in the two accessions (Figure 2C). Thus, approach herkogamy in natural *A. thaliana* accessions has a complex genetic basis and may have evolved independently multiple times. As a first step towards harnessing the power of *A. thaliana* for a molecular dissection of approach herkogamy, we here analyzed the developmental characteristics, environmental control, genetic architecture, and the effects of this trait on the mating patterns. Detailed analysis of molecular evolutionary mechanisms of approach herkogamy seems promising and must await further study.

## Materials and Methods

### Plant material and growth conditions

Detailed information about the *A. thaliana* accessions used in this study is listed in Table S1. Seeds of Col-0 (N1092), Bay-0 (N954), Sha (N929), and a male-sterile accession (N75) were obtained from the European Arabidopsis Stock Centre (http://arabidopsis.info/). Seeds were stratified at 4°C for 4–5 days to break seed dormancy, and were germinated and grown at 22/20°C (day/night) under long-day light conditions [16 h/8 h (day/night)] for 7 days. All plants were then vernalized at 4°C under short-day light conditions [8 h/16 h (day/night)] for 4–5 weeks and subsequently transplanted into individual pots and used for the experiments.

The expression of herkogamy is environment-dependent, and details of the growth conditions are therefore provided here. The pollinator-free greenhouse compartments used received natural light, supplemented by artificial light under low illumination, and had temperature/humidity control systems. We used long-day light conditions and 22/20°C (day/night) for plant growth in the greenhouse. Only artificial light was available in climate chambers with temperature/humidity control systems.

### Measurement of herkogamy

We measured anther height and stigma height in a freshly opened flower under a microscope (Figure S1A), and the degree of separation between anther and stigma (SAS, as the proxy of herkogamy) was estimated as the difference between stigma height and anther height (i.e., SAS  =  stigma height – anther height). For each individual, three to five freshly opened flowers were measured and for each genotype, at least three individuals were typically measured.

To assess changes in SAS during floral development and to determine the optimal stage for measurement, we first estimated SAS in flowers of Col-0, BRA, and SIM grown in a climate chamber at various developmental stages. For each inflorescence, we measured 6 flowers simultaneously and assigned the most recently opened flower to stage 0 (approximately equal to stage 13 described in [42]); three older flowers were consecutively assigned to stages 1, 2, and 3, respectively; whereas 2 younger flowers were assigned to stages -1 and -2. For each accession, mean SAS values were determined by measuring flowers from 6–7 inflorescences in 3 individuals. A polynomial regression was fitted to the SAS values against different developmental stages. Our results indicate that SAS values peaked at stage 0 in each investigated accession (Figure S1B), and this stage was subsequently used for all measurements.

### Estimation of fertilization rates of specific siliques

To assess the fertilization rate of a specific silique, we dissected the silique under a microscope and manually counted the numbers of fertilized and non-fertilized ovules (Figure S1C). The fertilization rate of each silique was calculated by dividing the number of fertilized ovules by the total number of ovules. The first 30 siliques along the main stems from three individuals of each accession (i.e., Col-0, BRA or SIM) were measured, and used for regression modeling and calculating overall self-fertilization rates (Figures 2A & 2B). In the assisted selfing and pollen siring groups, 15–40 siliques (mean  = 28) and 11–30 siliques (mean  = 24), respectively, were scored.

### Influence of ambient temperature on the expression of herkogamy

The effects of different ambient temperatures on herkogamy were tested in three climate chambers set to identical light conditions (i.e., long-day) at 16°C, 22°C, or 30°C. Eight replicates per accession (i.e., Col-0, BRA or SIM) were grown in a randomized design within each chamber. SAS was determined from three flowers per individual.

### QTL analysis

An F_2_ population, derived from a cross between SIM (mother) and Col-0 (father), was grown in the greenhouse. SAS was estimated in three flowers of each F_2_ individual, and the self-fertilization rate of each individual was estimated as the proportion of fertilized siliques (judged by the morphology of siliques) among the first 20 siliques along the main stem. In total, 227 F_2_ individuals were phenotyped and further genotyped using 47 fluorescent-labeled microsatellite markers (markers were selected from the following website based on their genomic positions: http://www.inra.fr/internet/Produits/vast/msat.php) distributed across the *A. thaliana* genome. Linkage maps were constructed using JoinMap 4 [43] and QTL analysis was performed with MapQTL 5 [44]. To improve the normality of phenotypic data, anther height and stigma height were box-cox transformed, while SAS was arcsin transformed. QTL mapping was initially performed on transformed data with interval mapping (IM) and followed by composite interval mapping, referred to as MQM mapping in MapQTL 5, where markers outside the test interval in the genome were used as cofactors to increase the power and precision of QTL identification. The significant cofactors for each MQM model were determined through an iterative automatic cofactor selection. The genome-wide logarithm of odds (LOD) significance threshold was obtained from permutation tests with 1000 replicates as implemented in MapQTL 5. The percentage of variance explained, and the additive and dominance effects of each QTL were estimated at the LOD peak using MapQTL 5.

### Field experiments at high and low altitudes

The field experiments were performed in the Swiss Alps (in the area of Flims, Switzerland) at one low altitude site (Boefel; 640m a.s.l.; 46°52′23.45′′N/9°31′07.01′′E) and one high altitude site (Nagens; 2170 m a.s.l.; 46°51′52.11′′N/9°14′11.83′′E). All necessary permits were obtained for these field studies: Boefel, permitted by Christian Gerber (contact address: Canalweg 21, 7023 Haldenstein, Switzerland. Tel: 0041 81 353 1493); Nagens, permitted by Laax-Gemeinde (contact person: Claudio Coray, Via Principala 91, 7031 Laax, Switzerland. Tel: 0041 81 921 4616). At each site, 130 plants from 15 accessions (Table S1; 8–9 replicates per accession) were randomized and transplanted into a rectangular block (filled with identical soil) with a distance of 10 cm between direct neighbors (Figure S2). Plants were watered 0–2 times in the first week after transplantation when necessary. Flowers on the main stems from healthy plants (from the 15^th^–25^th^ flowers) were chosen for measuring anther height and stigma height. Three flowers from three to five individuals of each accession were measured. Towards the end of the flowering season, all aboveground material of each plant was harvested, stored in a big envelope and dried in a climate chamber at 25°C for one week. Subsequently all seeds of each plant were collected.

### Common-garden experiment in a rural field site

The common-garden experiment was performed in an experimental station close to Zurich (47°27′00.41′′N, 8°40′57.22′′E; 550 m a.s.l.; No specific permissions were required because it is the experimental station of ETH Zurich and our plant materials were natural germplasms). The plants (i.e., Col-0, BRA, and SIM) were vernalized at 4°C for different time periods so as to synchronize flowering among the different accessions. Plants were then transplanted into large pots (35 cm in diameter) prior to flowering. In each pot, one test plant (Col-0, BRA, or SIM) was planted in the center and 5 plants with different genotypes were planted as potential pollen donors in a circle around the test plant at a distance of 10 cm (Figure S3). The pots were placed at random positions in grassland with distances of approximately 1.2 m among pots. After three weeks, all pollen-donor plants and unopened flower buds or inflorescences on the test plants were removed. Mature siliques were collected from each test plant.

### Estimation of outcrossing rates

We randomly chose 3–4 test plants from each accession out of each field site for estimating their outcrossing rates. For each test plant, 32–48 progenies were grown and genotyped at four microsatellite loci. For each multiplex set, four highly polymorphic makers labeled with different fluorescent dyes (i.e., FAM or HEX) were selected from 50 loci examined in all parental accessions (Table S2). Labeled PCR products were subjected to fragment analysis with a 3730xl DNA Analyzer (Applied Biosystems), and the results were analyzed using GeneMapper 4.0 (Applied Biosystems) software as follows: (**i**) for each individual, we first checked if there was a maternal allele for each of the four loci. If not, we regarded it as seed contamination and excluded it. Overall, we found very low seed contamination. (**ii**) We then examined if there were additional alleles to the maternal alleles. An individual offspring was regarded as outcrossed progeny (hybrid between two inbred accessions) if more than two additional alleles were present among the four investigated loci. Individuals with one additional allele, which was rarely observed in our samples, were considered possible technical errors, and were excluded from outcrossed progeny. (**iii**) To determine if we could effectively identify real outcrossed progeny for each outcrossed individual, we examined if the potential father could be found in one of the included accessions by comparing alleles. We analyzed 169 outcrossed individuals from 414 samples in details, and successfully identified potential fathers for 98.2% of the examined hybrids. Additional alleles in three exceptional individuals could be partially found in the studied accessions. This confirmed that real hybrid offspring were actually identified in our study. (**iv**) The outcrossing rate for each test plant was then estimated as the proportion of outcrossed progeny.

### Statistical analysis

Statistical analyses were performed using R [45]. Tukey's post-hoc significance tests were used for multiple comparisons, whereas Pearson's correlation tests were used to analyze trait associations. Two-way ANOVA was performed to assess the effects of genotype, ambient temperature and their interaction on SAS.

Non-linear least-square regression models for the self-fertilization rates of siliques along the main stems of the three studied accessions (i.e., Col-0, BRA, and SIM) were fitted in R. The function is: y  =  exp(a+b*x)/(1+exp(a+b*x)). *x* is a vector of predictors, indicating the position of a specific silique in the main stems, and *y* is the observation, i.e., the autonomous self-fertilization rate. *a* and *b* are regression parameters.

## Supporting Information

Figure S1 **Illustrations of the phenotypic measurements.**
(TIF)

Figure S2 **Illustration of the box design for the field experiments.**
(TIF)

Figure S3 **Illustration of the pot design for the common-garden experiment.**
(TIF)

Table S1 Information on *A. thaliana* accessions used in this study.
(XLSX)

Table S2 Information on the markers used in this study and their polymorphisms in the studied accessions.
(XLSX)
