# Supplementary figures and images for: Herkogamy and Its Effects on Mating Patterns in Arabidopsis thaliana
Source: PLoS One. 2013 Feb 26;8(2):e57902. doi: 10.1371/journal.pone.0057902 (PMC3582510; doi:10.1371/journal.pone.0057902)

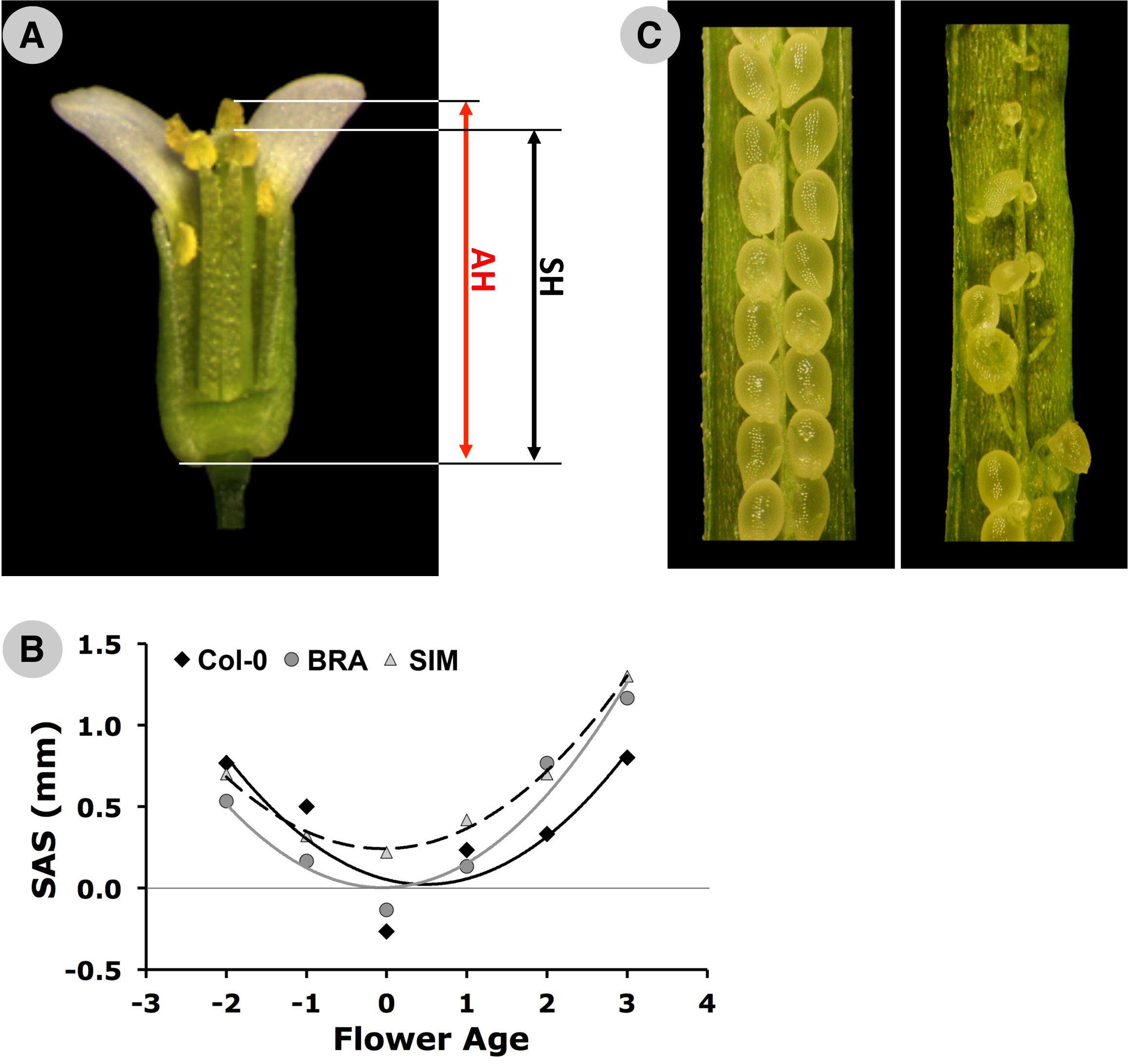

Supplement: Figure S1 — Illustrations of the phenotypic measurements. (TIF) [file pone.0057902.s001.tif]

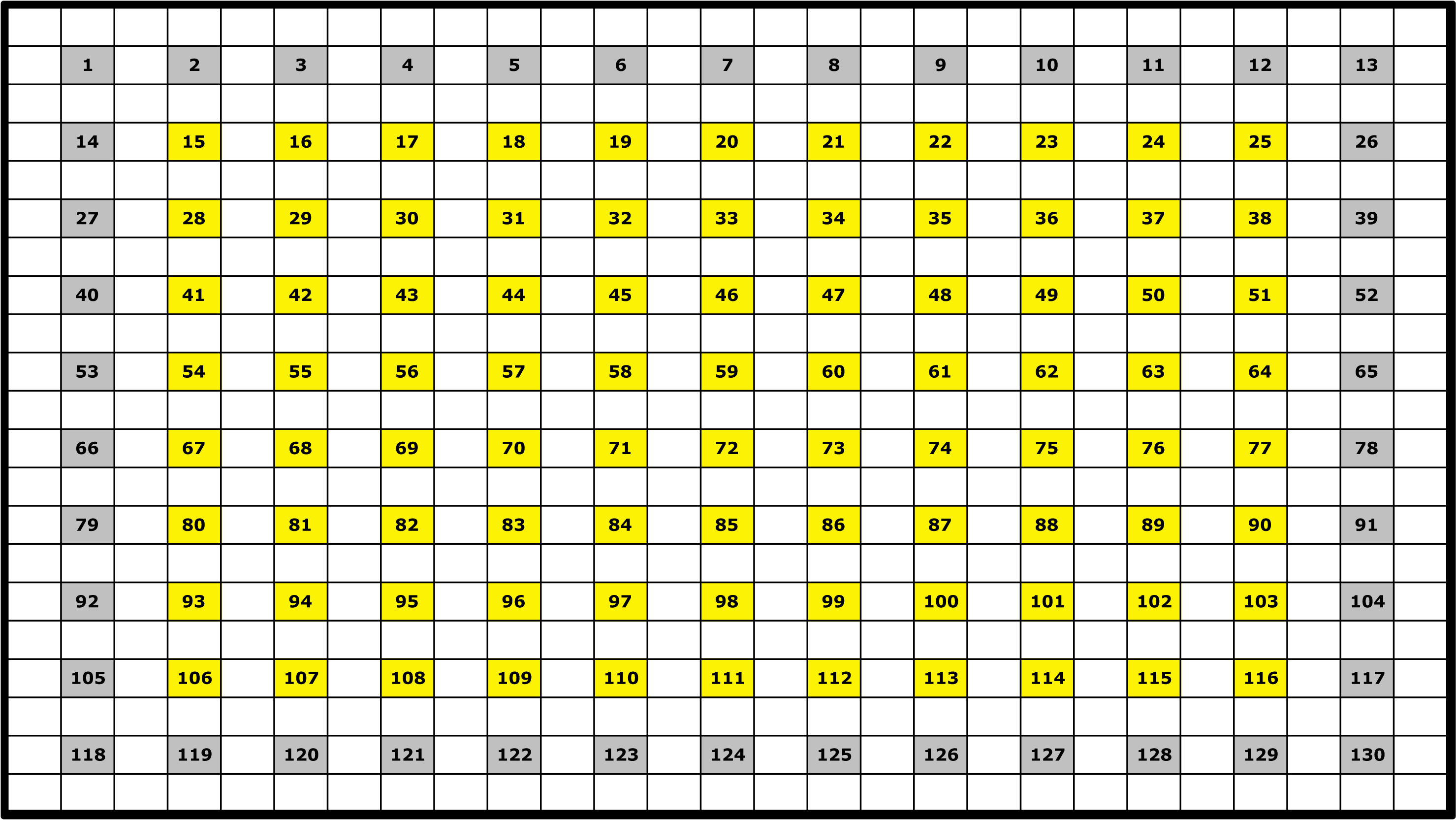

Supplement: Figure S2 — Illustration of the box design for the field experiments. (TIF) [file pone.0057902.s002.tif]

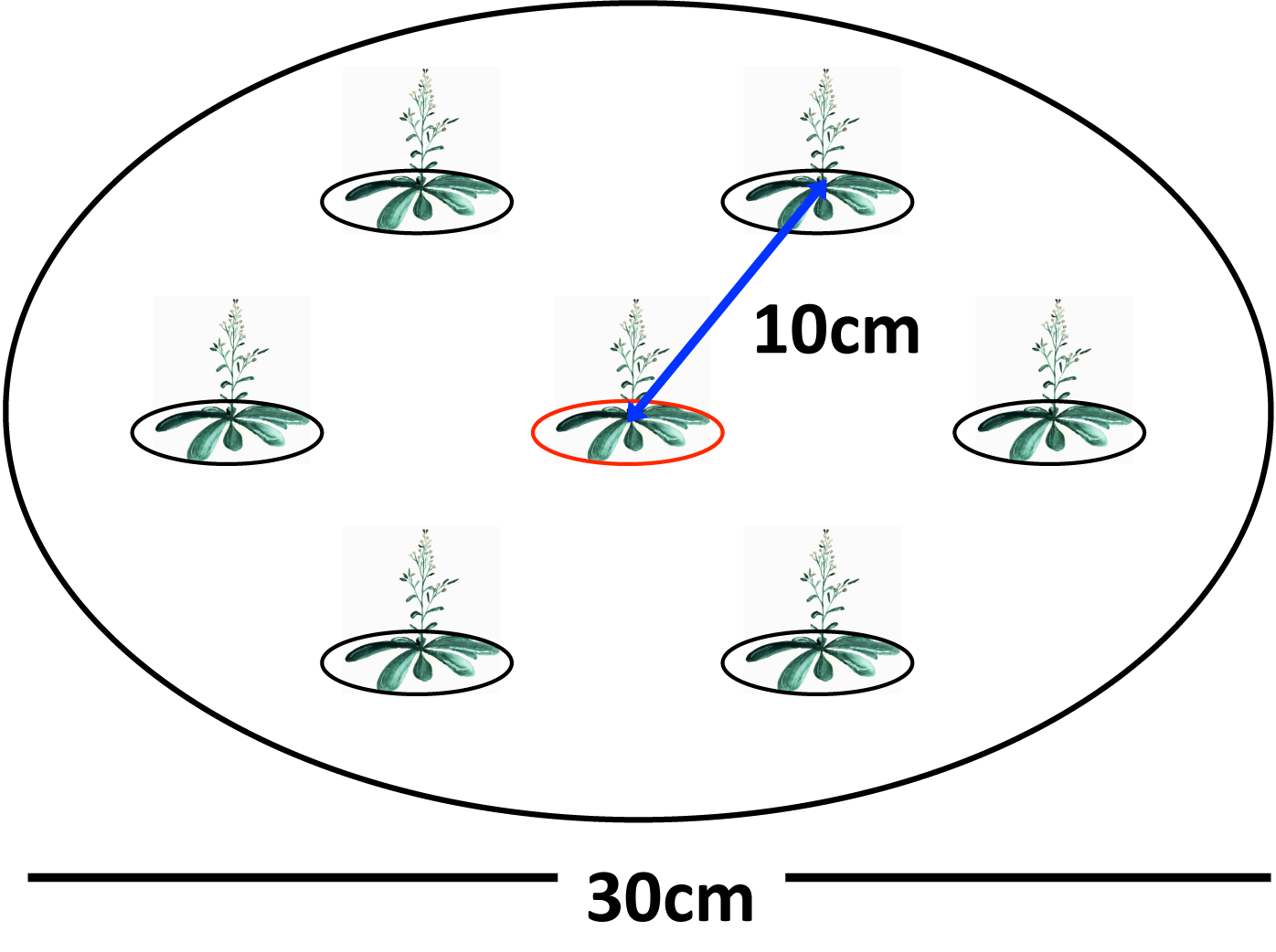

Supplement: Figure S3 — Illustration of the pot design for the common-garden experiment. (TIF) [file pone.0057902.s003.tif]
